# Our Contributors

**Published:** 1894-10

**Authors:** 


OUR CONTRIBUTORS.—This issue contains papers of practical interest, papers from which something may be learned. We have already referred elsewhere, briefly, to Dr. Fuller's paper. Dr. Cummings makes an argument for long ligatures and vaginal drainage, controverting Professor Paine's position taken in a discussion at the recent State Association meeting. Dr. Conerly tells much that is interesting in connection with climate in relation to consumption, and paints the charms of the historical Concho valley. Dr. Walker comes back at Dr. Oates, whose article appeared in the JOURNAL lately, and puts in evidence his experience, practical results from the use of calomel and quinine, so derided by Dr. Oates in malarial hæmaturia. Some of our bacteriological friends of the later editions,—the new issue of practitioners,—will read his paper with surprise, and will feel disposed, perhaps, to question his *doses*, forgetting that long ago that practice was taught by the ablest men. Fenner, for instance, was great on calomel and quinine in southern fevers; so was Drake; and Bemis, as cited by Dr. Walker. Let's see—1869–70? Twenty-five years, a quarter century. In that time what a change has taken place in the teachings of pathology, and consequently therapeutics? The germ theory was *born* since then (as were some of the doctors). Nevertheless, the pathology of malarial hæmaturia is not yet understood, and until it is, all treatment is more or less empirical; and whatever may be Dr. Walker's views of germ causation, and however far from "up to date" his treatment may seem, until somebody can show *better results* by another, he believes in sticking to the bridge he found would bridge him and his patient over. To think now, of giving dram doses of quinine till an ounce is taken in 24 hours, makes the younger generation's hair stand on end like quills of frightful porcupine. Dr. Sears, the Seer of Waco, our late President, stood up for the same kind of treatment, we believe, for typhoid fever, in a discussion in the Austin District Medical Society. While, as representing the other, or latter day school of practice, Dr. W. N. Rogers makes a plea for treatment based on the microbian pathology.

Well, doctors will differ. All this makes good reading, however, and variety is a necessity in reading as in diet.

Dr. Field's remarkable case of spinal bifida, upon which he operated with success, is also published herewith. Does any one know of a similar case on record anywhere? We do not.

